# Compulsory licensing of pharmaceuticals during public health crisis: a TRIPS framework analysis

**DOI:** 10.3389/fpubh.2025.1630586

**Published:** 2025-09-12

**Authors:** Zhanpeng Li, Peng Guo

**Affiliations:** ^1^Civil and Commercial Law School, Southwest University of Political Science and Law, ChongQing, China; ^2^Law School, Jinan University, Guangzhou, China

**Keywords:** TRIPS, compulsory licensing, waiver, public health, access to medicines

## Abstract

The COVID-19 pandemic has exposed inequities in global healthcare resource allocation, reigniting debates over international intellectual property (IP) protections. Among existing flexibility mechanisms, the TRIPS Agreement’s compulsory licensing provisions serve as a critical tool to ensure access to essential medicines. Member states can invoke Article 31(b)'s “national emergency” or “other circumstances of extreme urgency” clauses to utilize patented Pharmaceuticals for public health emergencies without prior authorization, a mechanism already effectively employed by multiple nations during the pandemic. However, due to procedural complexities and potential trade disputes associated with TRIPS, some developing countries have advocated for temporary IP waivers as an alternative solution. It must be emphasized that compulsory licensing retains unique value in balancing public health needs with IP protections. To better prepare for future health crises, improvements to the TRIPS compulsory licensing tool could focus on two priorities: prioritizing essential medicine accessibility over price control objectives, and enhancing local pharmaceutical production capabilities in developing nations.

## 1 Introduction

The COVID-19 pandemic has exposed substantial gaps in global vaccine distribution (1, 2), reigniting long-standing debates over whether intellectual property (IP) rights act as barriers to accessing affordable medicines (3). A nation’s true development hinges on the strength of its public health sector (4), with access to essential medicines serving as a core measure of developing countries’ capacity to deliver public health services (5). The *Declaration on the TRIPS agreement and public health* (hereafter *Doha Declaration*) reaffirms “the right of WTO members to use, to the full, the provisions in the TRIPS Agreement, which provide flexibility for this purpose” (6) to safeguard public health, particularly by advancing universal access to medicines (7). TRIPS Flexibilities refer to provisions within the TRIPS Agreement that allow WTO members to adapt IP rules to their national priorities and development needs when implementing the agreement into domestic law (8). Among these flexibilities, compulsory licensing stands out as a critical policy tool to curb patent abuses, lower drug prices, and ensure affordable access to essential medicines (9). During debates at the World Intellectual Property Organization (WIPO) assemblies, numerous developing countries within the WTO have recognized compulsory licensing as indispensable for maintaining economically viable pricing of vital medications (10).

Despite being established for decades, compulsory licensing has rarely been activated by countries in practice. The complexity of patents means that simply replicating production based on patent descriptions—even with significant financial and human investments—does not guarantee successful outcomes (11). However, compulsory licensing remains a critical legal right and a powerful negotiating tool (12). During the COVID-19 pandemic, multiple countries invoked Article 31 of the TRIPS Agreement to issue compulsory licenses for COVID-19 treatments, while the WTO adopted a specialized patent waiver proposal to address pandemic-related challenges (13). These novel applications of TRIPS flexibilities, while effectively safeguarding global public health during the crisis, have sparked renewed debates over IP protections (2). This study conducts a comparative analysis of TRIPS compulsory licensing and the IP waiver resolutions adopted during the COVID-19 pandemic, identifying persisting challenges and proposing practical solutions to better prepare for future public health crises.

## 2 Methodology

This study applies qualitative documentary analysis, adopting a critical narrative review framework to examine TRIPS Agreement-related materials (1995–2025) concerning public health crises—including legal documents, policies, cases, and scholarly publications. The research aims to establish an interdisciplinary framework integrating law, public health, and international relations, providing governance recommendations for future public health crises through the TRIPS lens.

### 2.1 Data sources and selection criteria

Laws and policies are primarily based on official WTO documents, selected for their authority and relevance. Key references include: I. The TRIPS Agreement, a cornerstone of WTO’s legal framework (14, 15); II. The *Doha Declaration*, the *Implementation of paragraph 6 of the Doha Declaration on the TRIPS Agreement and public health* (hereafter *General Council Decision*), and the *Amendment of the TRIPS Agreement*, which reflect WTO’s concrete actions in addressing medicine accessibility and public health crises (16); III. WTO Ministerial Conference decisions and member states’ formal proposals. As the organization’s highest decision-making body, the Ministerial Conference coordinates member positions, holds authority to amend or interpret TRIPS, and its resolutions capture the agreement’s latest developments (17).

Theory and practice supporting materials are drawn from a broad range of peer-reviewed journals, official and industry reports, and case studies, selected for their impact, timeliness, and diversity. These include: I. Key journals in public health and law, searched across databases such as PubMed, Scopus, Web of Science, HeinOnline, and Google Scholar. Primary search terms—including “TRIPS Flexibilities,” “compulsory licensing,” “intellectual property waiver,” “access to medicines,” “COVID-19,“and “pharmaceutical patents”—were used to capture the latest research developments on TRIPS health-related provisions; II. Official and industry reports from various countries, along with case studies on TRIPS implementation, were reviewed to reflect practical perspectives and compliance with the agreement. The selected reports and cases represent WTO member states with varying economic development levels and pharmaceutical capabilities, highlighting the real-world effects of TRIPS on health, economics, and politics (18).

### 2.2 Narrative approach

This study employs a critical narrative review approach, structured as follows: First, it examines the legislative background and implementation outcomes of TRIPS compulsory licensing and IP waivers, highlighting the comparative advantages of compulsory licensing. Subsequently, it analyzes the current challenges confronting the compulsory licensing system. Finally, it proposes targeted solutions to address these issues.

## 3 TRIPS flexibilities: a key legal tool for addressing global health crises

### 3.1 TRIPS compulsory licensing

In June 2001, the TRIPS Council convened its first dedicated session addressing intellectual property and public health, focusing on TRIPS-compliant access to medicines. This landmark discussion catalyzed the WTO’s adoption of three critical legal instruments to address public health crises: the *Doha Declaration*, the *General Council Decision*, and the *Amendment of the TRIPS Agreement*. Article 5 of the *Doha Declaration* unequivocally affirms that “each member has the right to determine what constitutes a national emergency or other circumstances of extreme urgency” for issuing compulsory licenses during public health crises (6). To better implement the TRIPS compulsory licensing system, paragraphs 2 and 3 of the *General Council Decision* waived TRIPS Article 31(f) and (h) obligations, enabling WTO members with pharmaceutical production capacity to export affordable medicines—via compulsory licensing—to developing and least-developed countries lacking such capabilities (19). These provisions were permanently codified into TRIPS through *Article 31bis* under the *Amendment of the TRIPS Agreement* (20). Collectively, these documents represent the WTO’s first substantive treaty revisions, legally and politically empowering developing nations to leverage compulsory licensing and parallel importation tools to enhance medicine accessibility.

During public health crises like the COVID-19 pandemic, member states can invoke the “national emergency” or “other circumstances of extreme urgency” provisions under Article 31(b) of the TRIPS Agreement to issue compulsory licenses for patented medicines or technologies (11, 21). Governments may use TRIPS Article 31 to authorize compulsory licensing for COVID-19 treatments, vaccines, and diagnostic tools, enabling rapid scaling of production (13). Multiple countries have already implemented such measures during the pandemic, including Hungary and Russia issuing compulsory licenses for Remdesivir, and Israel for Lopinavir/Ritonavir (22). Notably, even historically TRIPS-hesitant nations like Canada passed the *Act Respecting Certain Measures Related to COVID-19*, streamlining procedures to allow its government or designated entities to bypass patent protections when necessary to address public health emergencies (23). These actions demonstrate how TRIPS flexibilities can be mobilized during global crises to balance IP rights with urgent healthcare needs.

### 3.2 TRIPS IP waivers

The *Doha Declaration* outlines two mechanisms for enhancing medicine access during health emergencies: compulsory licensing and IP waivers (24). Article 9.3 of the *Marrakesh Agreement* empowers WTO Ministerial Conferences to waive treaty obligations with three-quarters majority approval under exceptional circumstances (25). In October 2020, India and South Africa proposed a landmark waiver proposal suspending 18 provisions across four TRIPS Agreement sections (copyrights, industrial designs, patents, and protection of undisclosed information) for COVID-19 prevention and treatment, with indefinite duration until global vaccine immunity (26). This sweeping proposal faced opposition from developed nations like the U.S., Switzerland, and the U.K. due to its broad scope and open-ended timeline (27, 28). Following revisions supported by African developing nations, India and South Africa narrowed their original proposal to focus exclusively on “health products and technologies” for COVID-19 diagnostics, therapeutics, and vaccines, while limiting the waiver’s duration to a minimum of 3 years from implementation (29). However, persistent disagreements over intellectual property coverage stalled consensus. By March 2022, WTO Director-General Ngozi Okonjo-Iweala facilitated quadrilateral negotiations among India, South Africa, the EU, and the U.S. (30), culminating in the *Ministerial Decision on the TRIPS Agreement* adopted at the 12th Ministerial Conference in June 2022. The final resolution restricts patent waivers to “production and supply of COVID-19 vaccines,” excluding treatments and medical devices. Notably, all developing country members are eligible for waivers, but country with vaccine production capacity are encouraged to voluntarily forgo these waivers through binding commitments. Eligible developing countries may use these flexibilities for 5 years, with annual WTO reviews and possible extensions (31).

### 3.3 Compulsory licensing vs. IP waivers

Countries advocating for IP waivers argue that the TRIPS compulsory licensing mechanism poses a barrier to accessing COVID-19 vaccines and treatments, as developing nations face institutional and legal difficulties in implementing it (26, 32). However, TRIPS flexibility mechanisms have proven both widely utilized and effective in practice. For instance, research shows that between 2001 and 2016, 89 countries invoked TRIPS flexibilities 176 times, with approximately 60% of these cases involving compulsory licensing. Over one-fifth of these applications specifically utilized transitional measures for pharmaceutical products in least-developed countries (33). The following discussion will highlight the critical role of compulsory licensing in promoting medicine accessibility.

- Legal feasibility. The waiver is not an inherent part of the TRIPS system but rather a temporary arrangement under the *Marrakesh Agreement* to address emergencies. As an exemption from international obligations, it is largely free from most TRIPS constraints but may easily conflict with national IP laws (Table 1). In contrast, compulsory licensing is an integral component of TRIPS, reflecting the objectives and principles of the IP system (34). Implementing compulsory licensing within the TRIPS framework ensures that patent usage aligns with its purpose. Through restrictions on authorized entities, scope, and duration, it strikes a balance between rights and obligations, maximizing societal welfare within the IP system (35). For example, Article 31(c) of TRIPS stipulates that “the scope and duration of such (compulsory licensing) use shall be limited to the purpose for which it was authorized,” ensuring its proper operation within the IP framework.
- Economic feasibility. Knowledge has the attributes of public goods—once it enters the public domain, it becomes nearly impossible to privately restrict others from accessing or using it (18). Therefore, IP waivers run counter to innovation by penalizing inventors who contribute to the public pool of knowledge (36–38). For instance, once a drug loses patent protection, low-cost generics can rapidly capture up to 90% of its sales (39). Meanwhile, drug development costs have skyrocketed with technological advancements. In 2019 alone, the U.S. pharmaceutical industry invested $83 billion in R&D—roughly 10 times the annual average in the 1980s (40). Unlike IP waivers, compulsory licensing offers a balanced solution (41). TRIPS provisions, particularly Article 31(f) on territorial restrictions and Article 31(h) on adequate remuneration, ensure that low-cost generic drugs do not disrupt other markets while guaranteeing fair compensation to pharmaceutical companies (Table 1), thereby sustaining innovation incentives.

**Table 1:** Main differences between the TRIPS Compulsory Licensing mechanism and the TRIPS Waiver Decision.

Factor	Issue	TRIPs CL	TRIPs Waiver	Implications
I. Legal feasibility	Authorizing entity	Authorized by domestic authorities, such as courts or intellectual property administrative agencies.	Decided by the Ministerial Conference, the highest authority of the WTO.	A blanket authorization by the WTO would be incompatible with national intellectual property laws.
	Scope of authorization	Authorization of such use shall be considered on its individual merits.	A categorical patent waiver that exempts entire classes of products or technologies.	A broad waiver would undermine intellectual property rights.
	Authorization conditions	Generally requires efforts to obtain authorization from the patent holder before issuing a compulsory license, but this requirement may be waived in cases of “national emergency” or “other circumstances of extreme urgency.”	Eligible member states are not required to obtain prior authorization from the rights holder.	Without oversight from rights holders, patents are prone to abuse.
II. Economic feasibility	Territorial restrictions	Primarily supply the domestic market, but exports are permitted to countries with limited pharmaceutical production capacity under the compulsory licensing scheme.	Eligible members may be exempt from the TRIPS Agreement Article 31(f) requirement that compulsory licensed products be predominantly supplied to the domestic market, and are permitted to re-export these products to other eligible member states.	Unrestricted re-export of waived patents would undermine patent holders’ market interests.
	Remuneration	Requires that right holders be paid adequate remuneration based on the economic value of the authorized use, taking into account the specific circumstances of each case.	Taking into account humanitarian and non-profit purposes to ensure equitable access to COVID-19 vaccines.	Setting prices at “non-profit” levels would severely undermine companies’ incentives for R&D.

## 4 Challenges of TRIPS compulsory licensing in health crises

While the TRIPS compulsory licensing plays a crucial role, it failed to deliver as expected during the COVID-19 pandemic. The reasons boil down to three factors: cumbersome legal procedures, political risks, and industrial capacity gaps.

### 4.1 Cumbersome legal procedures

The TRIPS compulsory licensing system involves a burdensome and arduous application process (42). Specifically, it requires both importing and exporting countries to issue parallel compulsory licenses, while mandating importing nations to prove their “insufficient manufacturing capacity”—a term lacking clear criteria under TRIPS (43). Additional administrative obligations, such as WTO notifications, further escalate compliance costs for exporting countries and deter generic drug producers from supplying developing and least-developed nations (11). Consequently, despite the *Doha Declaration*’s establishment of a parallel import system for pharmaceutical patents, only one successful case has materialized globally: Canada’s 2008–2009 export of 260,000 doses of generic HIV/AIDS drugs to Rwanda after Rwanda’s WTO declaration of production incapacity (44).

### 4.2 Political risks

Countries invoking compulsory licensing may risk WTO disputes or trade retaliation. For instance, the U.S. *Special 301 Report* and EU’s *Report on the Protection and Enforcement of Intellectual Property Rights in Third Countries* routinely scrutinize such measures. For example, when Thai authorities sought to exercise the compulsory licensing authority under the *Doha Declaration* to address public health needs, they faced accusations of “seizing property and violating trade rules.” In response to alleged violations of IP laws, the U.S. government placed Thailand on its “Special 301 Priority Watch List” (45). In another instance, the U.S. imposed punitive 100% tariffs on $390 million worth of Brazilian goods to coerce legislative changes to Brazil’s pharmaceutical patent laws (46).

### 4.3 Industrial capacity gap

While compulsory licensing grants legal access to patented technologies, it cannot substitute for the technical expertise required to manufacture complex pharmaceuticals (3, 11). In patent disclosures, companies often deliberately omit key manufacturing processes and procedures underlying their inventions. Although such ambiguity in information disclosure may stem from inherent language limitations (47), it is more common in the biopharmaceutical sector for firms to withhold manufacturing details to maintain a competitive advantage (48). Notably, trade secrets—critical for producing high-quality, safe, and effective drugs or vaccines—are exempt from compulsory patent licensing (49). For instance, despite Moderna’s pledge not to enforce its mRNA vaccine patents during the pandemic (50), other manufacturers failed to replicate the vaccine due to shortages of raw materials and inadequate manufacturing facilities (51). This demonstrates that even with waived IP restrictions, resource gaps and industrial barriers remain insurmountable hurdles for low- and middle-income countries (52).

## 5 Recommendations for TRIPS compulsory licensing to address future health crises

While many studies highlight the complexity of TRIPS compulsory licensing procedures (26, 32), the immediate challenge lies not in legal texts or processes, but in mitigating resistance from developed nations and strengthening domestic pharmaceutical capabilities. Therefore, reforms should prioritize two key dimensions: political strategy and industrial capacity.

### 5.1 Prioritizing essential medicine access over price control objectives

While compulsory licensing of pharmaceuticals has predominantly been associated with low- and middle-income countries seeking access to essential medicines like HIV treatments, developed nations have occasionally invoked this mechanism over the past two decades to address drug shortages and pricing concerns (53). For instance, Belgium’s parliamentary health committee recently debated legislation authorizing compulsory licensing to counter excessive drug pricing, tasking its national Health Care Knowledge Centre with analyzing the legal and economic feasibility of such measures (54). Similarly, U.S. Congress members proposed a 2018 bill empowering the government to issue compulsory licenses when pharmaceutical price negotiations for Medicare-covered drugs reach impasses (55, 56).

However, these actions risk undermining pharmaceutical innovation incentives in R&D-intensive economies (57). Industry pushback is exemplified by PhRMA’s stance in its Special 301 submissions, “American patients should not have to shoulder the burden of paying for global innovation. Compulsory licensing creates significant uncertainty for biopharmaceutical innovators and harms patients by undermining incentives for future research (58).” Therefore, neither the TRIPS Agreement nor the Doha Round negotiations have treated compulsory licensing as a tool for negotiating drug pricing or market access (7). Moreover, market practices demonstrate that competition and price negotiations can also drive down drug costs. Take AIDS medications, for example—between 1996 and 2001, the annual per-person treatment cost plummeted from around 10,000 to 295. This dramatic drop was not due to compulsory licensing but resulted from price competition between pharmaceutical companies and generic manufacturers, coupled with public pressure (59). In contrast, a study on the impact of compulsory licensing on antiretroviral drug prices in developing countries found that in 63% of cases, drugs produced under such patent authorizations were priced higher than the average procurement cost (60).

In a word, rather than pursuing price controls that provoke trade disputes, the compulsory licensing system should prioritize targeted humanitarian applications. This entails granting time-bound, crisis-specific patent access for essential medicines under TRIPS-compliant frameworks, ensuring affordability without destabilizing the innovation ecosystem that underpins long-term global health security.

### 5.2 Strengthening local medical production capacity

In the long term, the most effective defense against risks like WTO disputes or trade sanctions lies in strengthening a nation’s domestic medical production capabilities. The practical value of compulsory licenses diminishes when patented medications or vaccines lack viable alternatives (61). This reality is particularly acute in developing nations, where most lack the industrial infrastructure for independent, large-scale vaccine production and must rely on foreign support (62). To transform compulsory licensing from a legal tool into a practical solution, developing nations must couple legislative reforms with investments in local manufacturing infrastructure.

Addressing these challenges requires active collaboration with international organizations like WHO and UNCTAD, as well as specialized NGOs that provide both legal framework guidance and Substantial support (3, 63). A notable example is WHO’s COVID-19 Technology Access Pool (C-TAP), a global initiative facilitating access to IP, research data, and manufacturing know-how for COVID-related medical products (64). Early successes include developing nations acquiring COVID-19 serological antibody technology through this platform (65), demonstrating the potential of multilateral technical cooperation in bridging healthcare capability gaps.

Additionally, robust market demand is a prerequisite for nurturing a domestic pharmaceutical industry. To this end, conditional relaxation of TRIPS Article 31(f) territorial restrictions on generic drug sales could be considered. Before joining TRIPS, countries like India, Argentina, and Turkey successfully fostered thriving local pharmaceutical industries by either denying drug patents or imposing strict patent limitations—even Brazil’s weaker patent protections contributed to industry growth (66). Historically, even developed nations built their pharmaceutical sectors under weak patent regimes, with nearly all industrialized countries relying on technological imitation for early-stage accumulation (67). Specifically, exemptions from TRIPS Article 31(f) obligations could be granted to select least-developed and geographically proximate developing nations. This would both expand demand to lower generic manufacturers’ marginal costs and increase supply to reduce drug prices (68). For instance, third-world regions like East Africa could adopt a common market approach, implementing an integrated system for compulsory licensing, generic production, and distribution (69).

## Conclusion

Human and human rights must be at the center of pandemic prevention and control, with their protection should be considered of primary importance (70). In addressing public health crises, IP waivers lack legal and economic feasibility, making it only a temporary solution. A sustainable approach lies in improving the compulsory licensing system under the TRIPS framework. During the COVID-19 pandemic, TRIPS compulsory licensing revealed limitations in legal procedures, political risks, and production capacity, hindering its effectiveness. Moving forward, efforts should focus on both political and industrial dimensions—enhancing the TRIPS compulsory licensing system by ensuring access to essential medicines and strengthening local pharmaceutical production. In summary, this study helps clarify the role of compulsory licensing, safeguards the integrity and effective functioning of the TRIPS system, and enhances the TRIPS flexibility to better address future public health crises.

## Data Availability

The original contributions presented in the study are included in the article/supplementary material, further inquiries can be directed to the corresponding author.
